# Root coverage in class I gingival recession defects, combining rotated papillary pedicle graft and coronally repositioned flap, using a micro surgical approach: A clinical evaluation

**DOI:** 10.4103/0972-124X.51890

**Published:** 2009

**Authors:** Tella Asha Latha, Sabitha Sudarsan, K. V. Arun, Avaneendra Talwar

**Affiliations:** *Formally, Post Graduate Student, Department of Periodontics, Ragas Dental College and Hospital, Chennai, India*; 1 *Professor and Head, Department of Periodontics, Ragas Dental College and Hospital, Chennai, India*; 2 *Professor, Department of Periodontics, Ragas Dental College and Hospital, Chennai, India*; 3 *Lecturer, Department of Periodontics, Ragas Dental College and Hospital, Chennai, India*

**Keywords:** Class I gingival recession, coronally advanced flap, rotated papillary pedicle graft

## Abstract

**Background:**

The purpose of this case study was to evaluate the success and predictability of a rotated papillary pedicle graft in combination with the coronally advanced flap using surgical loupe (2.5X magnification) for the treatment of Miller's class I gingival recession.

**Materials and Methods:**

Fifteen systemically healthy patients with isolated gingival recession underwent the procedure. The probing depth, percentage root coverage, width of the keratinized gingiva and the gain in clinical attachment, papilla width, papilla height, area of the papilla at the donor site, were recorded at baseline, 3 months and 12 months.

**Results:**

All parameters except probing pocket depth, significantly improved from baseline to 12 months. The mean recession defect of 2.67 ± 0.03 mm present at baseline reduced to 0.13 ± 0.35 mm at the end of the 3^rd^ months and stabilized at 0.27 ± 0.59 mm at 12 months. The mean reduction in recession depth was 2.40 ± 0.03 mm at the end of the study. Complete recession coverage was obtained in 13 of the 15 (87%) of the cases treated with a mean percentage recession coverage at 12 months being 86 ± 35.19%. The gain in the width of the keratinized gingiva was 1.33 ± 0.13 mm at the end of the study. There was no postoperative morbidity from where the graft was harvested at the end of the study period.

**Conclusion:**

The use of magnification in mucogingival surgery resulted in achieving a high degree of success and predictability as well as an excellent esthetic outcome.

## INTRODUCTION

Soft tissue recession, defined as exposure of the root surface caused by an apical shift of the gingival margin, results in an unesthetic appearance, root hypersensitivity and root caries.[1,2] The treatment of choice for recession coverage should address the biological as well as the patients esthetic demands.[3] Amongst the various surgical techniques that have been advocated for the treatment of gingival recession the bilaminar technique has shown better root coverage predictability and esthetics.[4–6] The primary disadvantage of this procedure is the necessity of a second surgical site and postoperative morbidity.

Tinti and Parma- Benfananti[7] described a free rotated papilla autograft where connective tissue harvested from the papilla adjacent to the recession using a microsurgical approach was used for the management of multiple shallow recession defects. The advantage of this procedure was that a single surgical site was sufficient for both the recipient and donor site. The 180 degrees rotated free graft proposed by Tinti and Parma - Benfanti[7] required a bulk of papilla which might not be present in all recession sites. Additionally, the free graft may result in a compromised blood supply.

The present study utilized a modification of the technique proposed by Tinti and Parma- Benfananti,[7] where a pedicle graft was obtained from the connective tissue of the adjacent papilla, rotated 90 degrees from its recipient bed to cover the exposed root surface. The use of surgical loupes during a periodontal plastic surgical procedure is an economically viable tool that enable utilization of microsurgical instruments, enhances operator's visual acuity, allowing better manipulation and more accurate suturing of soft tissues.[8]

The aim of the present clinical study was to evaluate the predictability of the root coverage procedure (rotated papillary pedicle graft in combination with a coronally repositioned flap) when done with the aid of surgical loupes using microsurgical instruments.

## MATERIALS AND METHODS

### Patient selection

Fifteen patients in the age group of 20 -50 years who required root coverage in a single tooth either for esthetics or for treatment of sensitivity participated in the study. Patients were in excellent general medical health, with no detectable systemic contraindications to surgical treatment. The etiology of these multiple recessions was either due to plaque-induced gingival inflammation or incorrect brushing technique. Patients modified their brushing techniques, adopted the “roll technique” in the area to completely remove dental plaque without causing trauma to the exposed root surface and thin soft tissue and to improve soft tissue health prior to the surgical phase. All the patients agreed to participate in this study and a signed surgical consent form was obtained.

Mucogingival recession sites were selected on the basis of the following anatomic considerations proposed by Tinti and Parma- Benfananti;[7] 1) the width and depth of the gingival recession was less than or equal to 5 mm; 2) interproximal bone crests showed no periodontal lesion; 3) it is necessary to have thick and wide interproximal papillae not smaller than the recession defect; 4) it is not necessary to have residual keratinized tissue; and 5) deep gingival grooves were not present on the donor papillae.

Oral hygiene instructions were given to every patient to eliminate the etiological factors. Scaling and root planning were performed where indicated. Surgical treatment of the defect was not scheduled until the patient could demonstrate an adequate level of supragingival plaque control. Only patients with full mouth and local plaque score less than 20% and full mouth and local bleeding score of less than 15% in the index proposed by O'Leary *et al*.,[9] throughout the study period were included in the study.

### Clinical measurements

The following clinical measurements were taken at the facial aspect of the selected teeth one week before surgery (baseline) and repeated at three and 12 months postoperatively.

**Figure 1:**
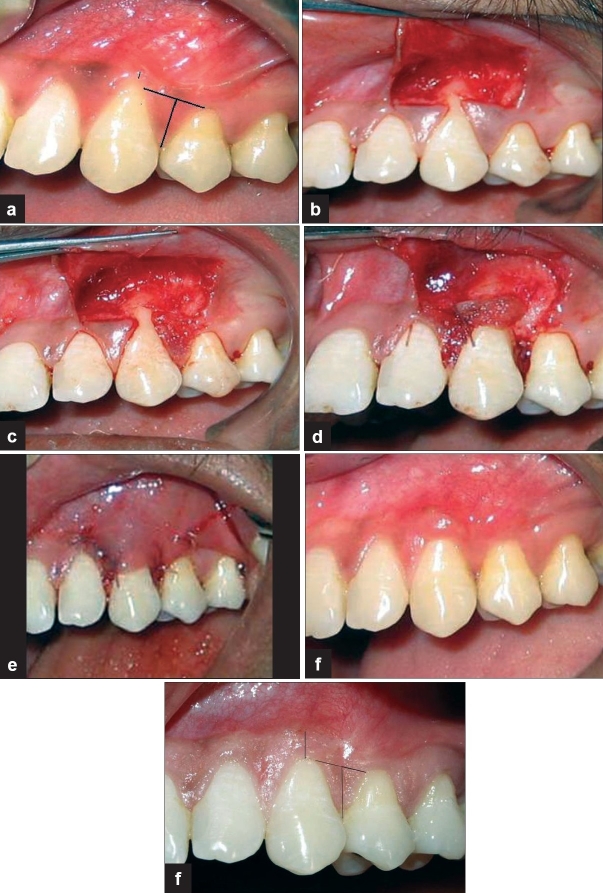
a) A Miller's class 1 gingival recession is present in correspondence to the upper canine, (Thin black line indicating width of keratinized gingiva; thick black line indicating length and width of the papilla). b) A trapezoidal partial thickness flap is raised. c) Distal papilla is de-epithelized. d) The de-epthelized papilla along with the periosteum attached to the bone is reflected and rotated 90 degrees to cover the recession defect. The papilla is sutured to the connective tissue bed. e) Flap coronally positioned to cover the graft. f) Three months after treatment. g) Complete root coverage and excellent esthetics at 12 months postoperatively.

- Gingival recession depth (RD), measured from the cemento enameljunction (CEJ) to the most apical extension of the gingival margin;
- Gingival recession width (RW), measured at the level of the CEJ;
- Probing depth (PD), measured from the gingival margin to the bottom of the gingival sulcus;
- Clinical attachment level (CAL), measured from the CEJ to the bottom of the gingival sulcus;
- Width of the keratinized gingiva (WKT), measured from the gingival margin to the mucogingival line [Figure 1a]
- Thickness of papilla, measured using an endodontic file at the center of the papilla with an imaginary line connecting the apex of the papilla to a midpoint on the base of the papilla;
- Area of the mesial and distal papilla, measured by including the surface within the triangle formed by a line drawn at the base of the papilla and along the lateral aspect of the papilla;
- Papilla height, measured as the perpendicular distance from the apex of the papilla to a imaginary line connecting the CEJ of the teeth adjacent to the papilla [Figure 1a];
- Papilla width, measured as the distance of the line connecting the CEJ of the involved tooth and the CEJ of the mesial or distal adjacent tooth [Figure 1a].

A single investigator took the clinical measurements. All measurements were taken by means of a William's periodontal probe (Hu- Friedy).

### Surgical technique

Patients were treated using minimally invasive surgical technique with the aid of a surgical loupe having a 2.5× magnification (Heinz, Germany). A sharp incision was performed at a 90 degree angle to the vestibule, following the mucogingival line, and extending in a mesio-distal direction to completely include the mesial and distal papillae adjacent to the gingival recession that needed to be treated. Sharp dissection was accomplished to create a partial thickness envelope flap on the underlying alveolar mucosa. Vertical releasing incisions were used at the line angles of the adjacent teeth to facilitate positioning the flap slightly coronally. The muscle fibers were dissected with a corono-apical direction of the blade well beyond the mucogingival junction for approximately 10 to 12 mm in order to reflect a partial thickness flap on the facial aspect only [Figure 1b]. The epithelium from the facial aspect of the donor papillae was debrided with a surgical blade [Figure 1c]. The exposed root surface was root planed with curettes to remove bacterial contamination and rotary instruments to reduce root convexity if required. A third incision was made starting at the coronal aspect of the denuded papilla and extending apically with the blade held parallel to the tooth surface to obtain a partial thickness papilla that was attached to the periosteuim. Two vertical incisions were made on the periosteum at a distance equal to the width of the papilla to a distance of 2 mm and sharp dissection done to separate periosteum from the bone along with the split thickness papilla. The apical aspect of the periosteum was still attached to the underlying bone. The papilla along with the periosteum is rotated 90 degrees in such a manner that lateral aspects of the papilla is at the CEJ and the apex lying on the connective tissue bed to which it is sutured with restorable sutures (Ethicon monocryl 6/0) [Figure 1d].

To improve vascularization and facilitate the healing process, this previously raised partial-thickness envelope flap is coronally positioned to a point where it may completely cover the rotated papillae and is then secured into this new position utilizing with resorbable sutures (Ethicon monocryl 6/0) and interrupted sutures for the vertical releasing incisions [Figure 1e].

Digital pression was applied for five minutes. No periodontal surgical dressing was used.

### Postsurgical follow-Up

Antibiotic therapy (amoxicillin 500mg, TDS) and suitable analgesic was prescribed for six days. Chlorhexidine-mouth wash (0.12%) was prescribed for 14 days. Tooth-brushing was discontinued for the first two weeks in the surgical site. After the first week the area was gently debrided with a cotton swab. After the second week the patient was instructed to use a soft tooth brush with a roll-technique followed by a 60-second rinse with chlorhexidine-digluconate. At the end of the six-week healing period, the patient returned to the usual oral hygiene technique.

The treated sites were periodically evaluated postoperative at three [Figure 1f], 12 months [Figure 1g] and relevant measurements were taken.

### Statistical analysis

The difference between the preoperative baseline, three and 12-month postoperative measurements within each group were examined by the ONE- WAY ANOVA test. The clinical measurements analysised were; recession depth (RD), recession width (RW), width of keratinized tissue (WKT), clinical attachment level (CAL), probing depth (PD), papilla height, papilla area and thickness of papilla.

The percentage of root coverage was calculated using the following formula: $\frac{\left(\text{Preoperative recession -Postoperative recession depth}\right)}{\text{Preoperative recession depth}}\times 100$

## RESULTS

Throughout the study period the patients maintained a good standard of supragingival plaque control. No adverse events were recorded during the postoperative period. The mean values of variables measured at baseline, three and 12 months after surgery are summarized in Tables 1 and 2. Complete root coverage was obtained in 13 of the 15 treated sites (86.67 ± 35.19%) at the end of 12 months.

**Table 1:** Clinical measurements of the recession defect at baseline, three and 12 months (mean ± SD)

Parameters	Baseline	Difference			(Baseline - 12 months)
			
		3 months	12 months	*P* value
RD reduction					
RD (mm)	2.67±0.62	0.13±0.35	0.27±0.59	< 0.001**	2.4 ± 0.03
RW reduction					
RW (mm)	3.40±0.74	0.07±0.26	0.27±0.26	< 0.001**	3.13 ± 0.48
WKT gain					
WKT (mm)	3.27±0.70	4.40±0.83	4.60±0.83	< 0.001**	1.33 ±.13
CAL gain					
CAL (mm)	4.07±0.80	1.77±0.82	1.47±0.52	< 0.001**	2.6 ± 0.28
PPD reduction					
PPD (mm)	1.46±0.51	1.29±0.31	1.09±0.52	NS	0.37 ± 0.01

NS - Not statistically significant

**Table 2:** Clinical measurements of the donor papilla at baseline, three and 12 months (mean±SD)

Parameters	Base line	3 months	12 months	*P*_0-3_ value	*P*_0-12_ value
P. height (mm)	4.47±0.74	3.03±0.77	4.40 ±0.74	<0.001**	NS
P. width (mm)	4.40± 0.74	4.40± 0.74	4.40± 0.74	NS	NS
P. area (mm^2^)	10.07±3.01	6.88±2.57	9.93±3.05	<0.001**	NS
P. thickness (mm)	3.54±0.44	2.20±0.65	3.41±0.58	<0.001**	NS

** The overall comparisons were highly significant at 1% level (p>0.001), NS - Not statistically significant

### Recession site characteristics

At baseline the mean probing depth was 1.46 ± 0.51 mm that reduced to 1.29 ± 0.31 mm at three months; and 1.09 ± 0.20 mm at the end of 12 months [Table 1]. The overall comparisons were not significant at one per cent level (p less than 0.001).

The mean clinical attachment level showed improvement from 4.07 ± 0.80 mm at baseline to 1.77 ± 0.82 mm at three months; and 1.47 ± 0.52 mm at the end of 12 months. The overall comparisons were highly significant at one per cent level (p less than 0.001).

Mean width of keratinized tissue increased from 3.27 ± 0.70 mm at baseline to 4.40 ± 0.83 mm at three months; and 4.60 ± 0.83 mm at the end of 12 months. The overall comparisons were highly significant at one per cent level (p less than 0.001).

At baseline, mean recession depth was found to be 2.67 ± 0.62 mm that reduced to 0.13 ± 0.35 mm at the end of three months; and stabilized at 0.27 ± 0.59 mm at the end of 12 months. The overall comparisons were highly significant at 1% level (*P* < 0.001).

At baseline, the mean recession width was found to be 3.40 ± 0.74 mm that reduced to 0.07 ± 0.26 mm at the end of three months; and was 0.27 ± 0.59 mm at the end of 12 months. Similar to RD, the overall comparisons were highly significant at one per cent level (p less than 0.001).

The overall percentage of root coverage considering the reduction in recession depths at different time intervals was found to be 94.44 ± 15.00% at three months; 86.67 ± 35.19% at the end of 12 months.

### Papillary characteristics

At base line, the thickness of papilla was 3.54 ± 0.44 mm that reduced to 2.20 ± 0.65 mm at three months, but later gained in thickness, increasing to 3.41 ± 0.58 mm at the end of 12 months [Table 2]. At base line, the mean papillary area was 10.07 ± 3.01 sq mm that reduced to 6.88 ± 2.57sq mm at three months, but later increased substantially to 9.93 ± 3.05 sq mm at the end of 12 months. At baseline mean papillary height was found to be 4.47 ± 0.74 mm that reduced to 3.03 ± 0.77 mm at the end of three months, and then increased to 4.40 ± 0.74 mm at the end of 12 months. The measurements recorded in the above papillary characteristic at 3 months were highly significant at one per cent level (p less than 0.001) but not significant at 12 months.

The mean preoperative papilla width was 4.7 ± 0.74 mm. The mean postoperative papilla width at three was also 4.7 ± 0.74 mm which remained unchanged till the end of the study.

## DISCUSSION

The purpose of this case study was to determine the effectiveness of a rotated papillary connective tissue pedicle graft used in combination with the coronally positioned flap as a root coverage procedure for Miller's class I recession. The major therapeutic goals of mucogingival surgery are esthetics, treatment of hypersensitivity and prevention root surface caries.[2] Miller[10] defined complete root coverage as the location of soft tissue margin at the CEJ, presence of clinical attachment to the root, sulcus depth of 2 mm or less and absence of bleeding on probing. Using this criteria for success, the subepithelial connective tissue graft described by Langer and Langer[11] in 1985, has become the ‘gold standard’ in the treatment of denuded roots. Since then, several techniques have described the use of the subepithelial connective tissue graft for root coverage and many have demonstrated high degrees of success ranging from 64.7-95.6%.[12,6,13] The use of a bilaminar technique has been found to be an effective method of achieving predictable and stable results in the treating isolated or multiple root recessions.

The surgical technique per se in any mucogingival surgical intervention has a major role in its successful outcome. The use of magnification devices along with microsurgical instruments facilitates the use of minimally invasive surgical technique that reduces tissue trauma. Studies by Tibbetts and Shanelec *et al*,[14] Belcher *et al*,[15] have addressed the advantages of using magnification in periodontal surgery. The effectiveness of the microsurgical approach for periodontal regeneration and root coverage has been reported by Cortellini *et al,*[16] Francetti *et al*,[17] etc. Magnification, illumination and increased precision in tissue manipulations result in minimal tissue damage during surgery with faster revascularization of the grafted tissue,[15] thus, minimizing the morbidity of the surgical procedure when compared with conventional surgical techniques.

The free rotated papilla autograft procedure is indicated in single Miller's Class I root recessions especially when a wide papilla is present in the mesial or distal aspect of the involved tooth. A clinical pilot study by Tinti and Parma-Benefenati[7] reported the successful use of this procedure in multiple recession cases and had achieving 91.87% coverage over a 12 months postoperative period. The limitation of this approach is related to the dimensions of the two papillae adjacent to the defect to be treated; which has to be match with the exposed root area. Thus, the papillary volume limits the application of this technique to only shallow gingival recessions.

In the present study the technique proposed by Tinti *et al*,[7] was modified, such that, the papillary graft retained its blood supply as it remained attached to the periosteum and therefore improved graft survival rate.

The mean percentage root coverage and mean gain of CAL obtained in this study were 86.67 ± 35.19 % and 1.33 ± 0.13 mm respectively at the end of 12 months. In addition, the mean gain of 1.33 ± 0.13 was obtained for the width of the keratinized tissue (WKT). These results obtained in our study seem to be slightly inferior to the values reported by Tinti and Parma-Benefenati^7^ and Francetti *et al,*[17] after treatment of the recession with the free rotated papllia autograft combine with the coronally advanced flap. The reduced mean percentage root coverage obtained in this study was because of the 70% coverage obtained in the two cases, both of which had compromised recession site characteristics when compared to the rest of the treated sites.

Throughout the study period, patients maintained good plaque control and hence plaque did not have any influence on the final stable attachment that was achieved.

Amongst the papillary parameters that were recorded, the mean papilla width remained unchanged throughout the 12 months study period. The mean papillary height, area of papilla and thickness of papilla which had reduced at the end of three months gradually increased and by the end of 12 months complete fill of gingival embrasure was achieved.

The limitations of this method remains the same as that proposed by Tinti *et al*,[8] in that adequate papillary volume is essential for post surgical success. This method may not be applicable even for Miller's Class II recession due to this reason. However, the lack of a second surgical site and good post operative results achievable makes it a viable procedure for Miller's Class I recessions.

Within the purview of this study it may be concluded that a rotated papilla graft, when combined with a coronally advanced flap with the aid of microsurgical loupes and instruments, offers a successful and viable alternative for the coverage of localized gingival recessions, especially Millers class gingival recessions.
